# Association of contraception use and pregnancy intention with perinatal depression risk among Omani mothers—a longitudinal cohort study

**DOI:** 10.3389/fgwh.2025.1497698

**Published:** 2025-01-31

**Authors:** Atika Khalaf, Nawal Al Amri, Pernilla Ny, Rebecca Mathew

**Affiliations:** ^1^ The PRO-CARE Group, Faculty of Health Science, Kristianstad University, Kristianstad, Sweden; ^2^ Hind Bint Maktoum College of Nursing and Midwifery, Mohammed Bin Rashid University of Medicine and Health Sciences, Dubai Health, Dubai, United Arab Emirates; ^3^ Maternal and Child Health Department, College of Nursing, Sultan Qaboos University, Muscat, Oman; ^4^ Department of Health Science, Faculty of Medicine, Lund University, Lund, Sweden; ^5^ Instructor Nursing, Fatima College of Health Sciences, Ajman, United Arab Emirates

**Keywords:** pregnancy planning, contraception use, antenatal depression, postnatal depression, Oman

## Abstract

**Background:**

Unplanned pregnancy is significantly associated with an increased risk of perinatal depression (antenatal and postnatal depression), emphasizing its prevalence and its potentially detrimental effects on both maternal and child health. This study aimed to investigate the association of contraception use and pregnancy intention with the risk of perinatal depression among Omani mothers.

**Methods:**

A prospective longitudinal study design was employed to investigate perinatal depression risk in mothers attending antenatal health care services in Oman. Perinatal depression risk was assessed using the Edinburgh Postpartum Depression Scale during the third trimester and postpartum visits. Multiple linear regression analyses were utilized to explore relationships between the risk of perinatal depression and pregnancy-related factors, contraception use, and sociodemographic variables.

**Results:**

The study involved 300 participants with a mean age of 30.8 years (SD = 5.47). The majority of participants reported planned pregnancy (74.0%), no use of contraception (66.0%), and being multiparous (72.7%). A significantly higher proportion (87.8%) of women with planned pregnancies were primiparous (*p* < 0.001). Besides family structure (core family, *p* = 0.025) and monthly income (1,000 OMR or below, *p* = 0.021), mothers who were pregnant for the first time (*p* < 0.001), and those who were primiparous (*p* < 0.001) did not use contraception. The regression models showed a significant association between the antenatal and postnatal depression scores (*p* < 0.001, 95% CI 0.401–0.603) according to the Edinburgh Postpartum Depression Scale.

**Conclusions:**

The findings suggest that women with unplanned pregnancies warrant attention for early detection and preventive interventions, irrespective of their emotional stance. Incorporating routine mental health screening into perinatal care can facilitate early detection, and targeted interventions, contributing to improved maternal mental well-being.

## 1 Introduction

Approximately 121 million pregnancies globally each year, accounting for nearly half, are unplanned (1), contributing to negative health outcomes for women and children (2, 3). In the context of women's mental health, the impact of unintended pregnancy has garnered attention due to its potential adverse effects including antenatal depression (4, 5), and postnatal depression (PPD) (6).

According to Centers for Disease Control and Prevention, unplanned pregnancy could result from mistimed pregnancies or when one or both partners perceive the current pregnancy as unwanted (7). Pregnancy planning is one of the most researched factors, with multiple and recent studies indicating that unplanned pregnancy is a risk factor for perinatal depressive symptoms (PND) (8, 9). The Arab region faces a tangible challenge in addressing unplanned pregnancies, particularly among a considerable proportion of married women of reproductive age who still express an “unmet need” for family planning (10). Decreasing unplanned pregnancies not only enhances women's reproductive well-being but also contributes to the reduction of maternal and neonatal mortality and morbidity (11, 12).

Although unplanned pregnancies have been identified as having adverse effects on child health, cognitive development, and birth outcomes (13), limited research has explored the connection between unplanned pregnancies and the risk of PPD within Middle Eastern contexts. The available literature primarily focuses on the social, economic, and health consequences of unplanned pregnancies, as well as the factors influencing women's adoption and continuation of contraceptive methods (13, 14). A study delved into the correlation between birth intention and the likelihood of PPD, utilizing data from the Young Lives Study (YLS) first wave conducted in 2002 across Ethiopia, Peru, India, and Vietnam (15). The researchers observed that mothers reporting unwanted pregnancies in each nation faced a significantly higher risk of PPD compared to those with planned pregnancies. Consequently, strategies aimed at reducing the incidence of unplanned pregnancies could play a pivotal role in mitigating the prevalence of PPD among mothers in these countries (15).

As recent studies have shown that unplanned pregnancy is significantly associated with PPD, reasons discussed indicate that women lacking preparedness financially, mentally, or socially to cope with the responsibilities of pregnancy may be susceptible to developing antenatal depression or PPD (16–18). Furthermore, in such situations, these women may encounter difficulties in balancing maternal responsibilities with concurrent obligations at home and work. Another contributing factor could be the presence of unstable psychosocial circumstances, leading to decreased feelings of security and attachment to their partners (18). Moreover, couples facing unplanned pregnancies are more prone to encountering marital problems, thereby elevating the risk of antenatal depression (19).

According to the 2019 National Health Survey in Oman, only 24% of married women of reproductive age used contraception (20). The antenatal care coverage exhibited a significant and consistent decline from 90% in 2015 to 83% in 2019. Furthermore, in 2019, approximately 74% of pregnant women attended antenatal care more than four times during their pregnancy (20). Moreover, the lack of routine screening for maternal mental wellness during pregnancy underscores the importance of identifying predictors of PND. The findings on the relationship between contraception use and antenatal depression contribute to the broader discussion on early detection and intervention. Although this study does not focus on screening and interventions, it highlights the need for comprehensive approaches to maternal mental health care, including routine screening, to ensure timely identification and support for mothers at risk of PND (21).

Despite the recognized association between unplanned pregnancies and PND, the research gap lies in the limited exploration of this connection in Oman. Upon reviewing the available literature, only one study was identified focusing on risk factors for antenatal depression, rather than specifically addressing pregnancy intention and contraception use in relation to PND (22). Therefore, the present study aims to address this gap by investigating the correlation between pregnancy intention and the risk of developing PND (i.e., both antenatal and postnatal depression). We believe that this research will provide valuable insights into the relationship between contraception use, pregnancy intention, and the risk of PND among Omani mothers.

## 2 Materials and methods

### 2.1 Study design

Using a quantitative approach, a prospective longitudinal study design was employed to address research questions related to PND risk. Mothers attending antenatal health care services were screened for PND risk at two time points—first during the third trimester visit (antenatal depression) and then at the postpartum visit to the same clinic. The Edinburgh's Postpartum Depression Scale (EPDS) used as the tool for assessing PND risk, is known for its widespread use and demonstrated high sensitivity (86%) and specificity (78%), and positive predictive value (73%) for use in pregnant and postpartum women from across diverse backgrounds and cultures (23, 24). EPDS consists of ten Likert-scale items, graded from 0 to 3 that evaluate emotional experiences over the past seven days. The total score is the sum of all ten items, i.e., 0–30 points with higher scores indicating a higher risk of maternal depression. Although different threshold scores have been used in validation studies, ranging from 9 to 13 points, we followed the existing recommendation of a score of 9 or more indicating an increased risk for maternal depression (25–27).

Additionally, sociodemographic data, including maternal age (continuous variable), pregnancy status (planned/unplanned pregnancy), contraception use (yes/no), parity (primi/­multiparous), gravida (1–5 or more), employment status (yes/no), accommodation (own/shared house), family structure (core/compound family), and household's monthly income (<500, 500–1,000, >1,000 Omani Riyals), were collected as self-reported by the participants.

### 2.2 Sample and context

The participants were volunteers recruited from the waiting rooms of three major healthcare centers in Wilayat Ibri in the northeastern part of the Sultanate of Oman. Both healthcare centers and participants were selected conveniently to reach the required sample size of 289 based on a 5% measuring error, an estimated prevalence of 25% (based on previous evidence suggesting that the prevalence of antenatal depression can range from 10% to 30% (28), and following the sample size equation: *n* = *Z*^2 ^ ×  *P*×(1-*P*)/d^2^. The main selection criteria for eligible participants were mothers aged 18 years or older, who were fluent in Arabic or English, and who did not have a self-reported diagnosis of depression. The exclusion criteria included mothers with intellectual disabilities that prevented them from effectively communicating their responses.

### 2.3 Data collection

One midwife from the respective healthcare centers assisted in the recruitment and data collection processes after receiving individual training on how to obtain written informed consent and use the screening instrument, i.e., EPDS. The participants were recruited from the waiting rooms of the involved healthcare centers during their visit to the antenatal clinic. The same mothers were then asked to report their mental health status by taking the survey again during their postpartum visit (2–6 weeks after childbirth). Data collection was initiated on June 15, 2022, and finalized in November of the same year. Mothers were screened twice: once during pregnancy and once at postpartum visit. The recruitment, data collection, and data entry processes were conducted concurrently with these screenings. Mothers were asked to complete the survey, including the sociodemographic data, individually or through individual interviews. In both cases, data were self-reported using hard copies of the survey. Pregnancy intention was measured in a close-ended question about whether the current pregnancy was planned or not.

A total of 300 mothers participated in the initial phase of the project and were screened for risk of antenatal depression as well as complete sociodemographic data reporting. At the follow-up phase during the postpartum visit, a drop-out of 37 participants was observed; hence, the postpartum data is based on 263 participants.

### 2.4 Data analysis

The initial phase of data analysis involved descriptive procedures, encompassing normality tests and assessments of assumptions for subsequent statistical tests. Participant characteristics were summarized during this phase.

The subsequent phase employed inferential statistics, employing group comparisons (Chi-square) and multiple linear regression analyses to assess the relationships between pregnancy intention (Planned/unplanned pregnancy), contraception use (Yes/No), and sociodemographic factors. The dependent variables included antenatal and postpartum EPDS scores, with pregnancy intention and contraception use as independent variables. Other variables entered in the linear regression model were age, postpartum EPDS, planned pregnancy, contraception use, employment status, accommodation, family structure, monthly income, gravida, and parous as independent variables. Dummy variables were created for categorical variables with more than two categories. Both linear regression models demonstrated a significant overall fit (ANOVA *p*-value <0.001). The significance level of all statistical analyses was set at *p*-value <0.05. All analyses were conducted using IBM SPSS Statistics version 28.

### 2.5 Ethical considerations

The project obtained approval from two ethical committees, namely the College of Nursing, the Medical Ethics Committee at Sultan Qaboos University (REF. NO. SQU-EC/­434/2021 – MREC #2426), and the Ministry of Health of Oman (MH/DGHS/DG/212­3111­067), before commencing data collection. Participating mothers were provided comprehensive written and verbal information about the project, including its objectives, methods, and the researcher's contact details (NAA), before signing a written consent form. Mothers were encouraged to reach out to healthcare centers for any signs of depression, but no mothers contacted the healthcare centers regarding their psychological status. The study adhered to the principles outlined in the Helsinki Declaration, and signed informed consent was obtained from all participating mothers (29). The participants were guaranteed confidentiality, and anonymity, and that they were free to participate and withdraw from the study without any reason or need for explanation and with no risk of facing consequences.

## 3 Results

### 3.1 Socio-demographic characteristics

The socio-demographic profile of the participating mothers is detailed in Table 1. The participants’ ages ranged from 18 to 46 years, with a mean age of 30.8 years (SD = 5.47). EPDS scores indicated an average antenatal score of 9.13 (SD = 5.25) and a postpartum score of 9.18 (SD = 5.00), with one missing case in antenatal EPDS and 38 missing cases in postpartum EPDS.

**Table 1:** Socio-demographic characteristics of the participants (*n* = 300).

Variable	m (SD)	Min-max
Age	30.8 (5.47)	18–46
Antenatal EPDS^b^	9.13 (5.25)	0–27
Postpartum EPDS^c^	9.18 (5.00)	0–23
	Frequency (*n*)	Valid percentage (%)
Planned pregnancy		
Yes	222	74.0
No	78	26.0
Contraception use		
Yes	102	34.0
No	198	66.0
Parous		
Primiparous	82	27.3
Multiparous	218	72.7
Gravida^d^		
1	74	24.7
2	49	16.3
3	60	20.0
4	49	16.3
5 or more	68	22.7
Employment		
Employed	91	30.3
Unemployed	209	69.7
Accommodation		
Own house	149	49.7
Shared house	151	50.3
Family structure		
Core family	168	56.0
Compound family	132	44.0
Monthly income		
<500 OMR^a^	84	28.0
500–1,000 OMR	167	55.7
>1,000 OMR	49	16.3

^a^ OMR, Omani Riyal, EPDS, Edinburgh postpartum depression scale.

^b^ Antenatal EPDS had 1 missing case.

^c^ Postpartum EPDS had 38 missing cases.

^d^ Gravida is all pregnancies including those ended in miscarriage and abortion.

A substantial majority reported planned pregnancies (*n* = 222, 74.0%), no use of contraception (*n* = 198, 66.0%), and being multiparous (*n* = 218, 72.7%). Gravidity varied, with 24.7% (*n* = 74) having one child and decreasing percentages for higher numbers of children. Employment status indicated that 30.3% (*n* = 91) were employed, while the housing arrangements were evenly split between owning a house (*n* = 149, 49.7%) and residing in a shared house (*n* = 151, 50.3%). The majority of the mothers were living in core families (*n* = 168, 56.0%) with a monthly income of 500–1,000 OMR (*n* = 167, 55.7%) (Table 1).

### 3.2 Association of socio-demographic factors with pregnancy planning and contraception use

Table 2 illustrates the comparison between women with planned and unplanned pregnancies, as well as those who used contraception vs. those who did not concerning their socio-demographic characteristics. Statistically significant differences were found between pregnancy planning and various socio-demographic variables. The analysis showed a significantly higher proportion of mothers with unplanned pregnancies among non-users of contraception (*p* < 0.001). In addition, a significantly higher proportion of women with planned pregnancies were primiparous (87.8%) compared to those with unplanned pregnancies (*p* < 0.001). Moreover, significantly more mothers planned their pregnancies regardless of how many times they had been pregnant previously (*p* < 0.001).

**Table 2:** Group comparisons of women with planned/unplanned pregnancy and those who used/did not use contraception.

Variable	Planned pregnancy, *n* = 300		*p*-value	Use of contraception, *n* = 300		*p*-value
	Yes, *n* = 222 (74.0%)	No, *n* = 78 (26.0%)		Yes, *n* = 102 (34.0%)	No, *n* = 198 (66.0%)	
Planned pregnancy						**<0**.**001**
Yes				63 (28.4)	159 (71.6)	
No				39 (50.0)	39 (50.0)	
Contraception use			**<0**.**001**			
Yes	63 (61.8)	39 (38.2)				
No	159 (80.3)	39 (19.7)				
Parous			**<0**.**001**			**<0**.**001**
Primiparous	72 (87.8)	10 (12.2)		7 (8.5)	75 (91.5)	
Multiparous	150 (68.8)	68 (31.2)		95 (43.6)	123 (56.4)	
Gravida			**<0**.**001**			**<0**.**001**
1	65 (87.8)	9 (12.2)		6 (8.1)	68 (91.9)	
2	35 (71.4)	14 (28.6)		15 (30.6)	34 (69.4)	
3	50 (83.3)	10 (16.7)		24 (40.0)	36 (60.0)	
4	30 (61.2)	19 (38.8)		31 (63.3)	18 (36.7)	
5 or more	42 (61.8)	26 (38.2)		26 (18.2)	42 (81.8)	
Employment			0.339			0.585
Employed	64 (70.3)	27 (29.7)		33 (36.3)	58 (63.7)	
Unemployed	158 (75.6)	51 (24.4)		69 (33.0)	140 (67.0)	
Accommodation			0.391			0.517
Own house	107 (71.8)	42 (28.2)		48 (32.2)	101 (67.8)	
Shared house	115 (76.2)	36 (23.8)		54 (35.8)	97 (64.2)	
Family structure			0.857			**0**.**025**
Core family	125 (74.4)	43 (25.6)		48 (28.6)	120 (71.4)	
Compound family	97 (73.5)	35 (26.5)		54 (40.9)	78 (59.1)	
Monthly income			0.174			**0**.**021**
<500 OMR^a^	60 (71.4)	24 (28.6)		31 (36.9)	53 (63.1)	
500–1,000 OMR	130 (77.8)	37 (22.2)		47 (28.1)	120 (71.9)	
>1,000 OMR	32 (65.3)	17 (34.7)		24 (49.0)	25 (51.0)	
Antenatal EPDS^b^	9.04 (5.14)	9.37 (5.56)	0.633	9.50 (5.43)	8.93 (5.16)	0.375
Postpartum EPDS^b^	9.19 (5.18)	9.14 (4.52)	0.942	9.17 (4.91)	9.18 (5.06)	0.987

Statistically significant values (*p* < 0.05) are bold.

^a^ OMR, Omani Riyal.

^b^ Values presented in means and SD.

As for the use of contraception, significantly more mothers living in core families (i.e., nuclear family unit, which includes the parents and their children) (*p* = 0.025) did not use contraception. In addition, significantly more mothers with an income of 1,000 OMR or below (*p* = 0.021), those who were pregnant for the first time (*p* < 0.001), and those who were primiparous (*p* < 0.001) did not use contraception.

The mean antenatal EPDS scores for mothers with planned pregnancies and those with unplanned pregnancies were 9.04 and 9.37, respectively. However, this difference was not statistically significant (*p* = 0.633). Similar results were observed in the postpartum period, where no significant differences were found between the two groups. Additionally, the mean EPDS scores did not differ significantly between mothers who used contraception prior to their current pregnancy and those who did not, in both the antenatal (*p* = 0.375) and postpartum periods (*p* = 0.987).

### 3.3 Predictors of antenatal and postpartum depression risk

The regression analysis revealed that none of the variables were significantly associated with antenatal depression risk (Table 3). Similarly, in the postpartum period, no variables were found to be significant predictors of postpartum depression risk, with the exception of antenatal EPDS scores. Antenatal EPDS scores were significantly associated with postpartum EPDS total scores (B = 0.51, *p* < 0.001, Beta = 0.52) (Table 4). The Beta value of 0.51 indicates that for every one-unit increase in the antenatal EPDS score, the postpartum EPDS score is expected to increase by 0.51 units, holding all other variables constant. This suggests a positive and significant relationship between antenatal and postpartum depression scores, implying that higher levels of depressive symptoms during pregnancy are associated with higher levels of depressive symptoms postpartum.

**Table 3:** Results of linear regression analysis between antenatal EPDS and selected variables.

Model	Unstandardized coefficients		Standardized coefficients	t	Sig.	95.0% Confidence interval for B	
	B	Std. Error	Beta			Lower bound	Upper bound
(Constant)	11.07	2.95	0.00	3.93	<0.001	5.26	16.88
Pregnancy planning	0.16	0.72	0.01	0.22	0.829	−1.27	1.58
Contraception use	−0.32	0.71	−0.03	−0.45	0.656	−1.72	1.09
Interaction variable^a^	2.18	1.45	0.35	1.50	0.134	−0.68	5.03
Maternal age	−0.12	0.08	−0.12	−1.54	0.125	−0.26	0.03
Number of children	1.34	0.85	0.12	1.57	0.117	−0.34	3.02
Parous	0,28	0.87	0.02	0.32	0.747	−1.43	1.99
Monthly income	0.47	0.63	0.04	0.76	0.449	−0.76	1.70
Family structure	−0.29	0.67	−0.03	−0.43	0.666	−1.60	1.03

^a^ Interaction variable = interaction between pregnancy planning and contraception use.

Note: Variables entered in the equation (Enter)—dependent variable: Antenatal EPDS, independent variables: planned pregnancy, contraception use, interaction variable, maternal age, number of children, parous, monthly income, and family structure. Model summary: r = 0.13, R^2^= 0.02, ANOVA *p*-value = 0.640.

**Table 4:** Results of linear regression analysis between postpartum EPDS and selected variables.

Model	Unstandardized coefficients		Standardized coefficients	t	Sig.	95.0% Confidence interval for B	
	B	Std. Error	Beta			Lower bound	Upper bound
(Constant)	6.90	2.69	0.00	2.56	0.011	1.60	12.20
Antenatal EPDS	0.51	0.05	0.52	9.75	<0.001	0.41	0.61
Pregnancy planning	0.14	0.62	0.01	0.22	0.827	−1.09	1.36
Contraception use	−0.40	0.62	−0.04	−0.65	0.519	−1.63	0.82
Interaction variable^a^	0.25	1.26	0.04	0.20	0.844	−2.22	2.72
Maternal age	0.05	0.06	0.05	0.71	0.480	−0.08	0.17
Number of children	−1.19	0.74	−0.12	−1.61	0.109	−2.65	0.27
Parous	−0.94	0.76	−0.08	−1.23	0.219	−2.44	0.56
Monthly income	0.11	0.54	0.01	0.20	0.845	−0.97	1.18
Family structure	−0.48	0.58	−0.05	−0.82	0.411	−1.62	0.67

^a^Interaction variable = interaction between pregnancy planning and contraception use.

Note: Variables entered in the equation (Enter)—dependent variable: Postpartum EPDS, independent variables: antenatal EPDS, planned pregnancy, contraception use, interaction variable, maternal age, number of children, parous, monthly income, and family structure. Model summary: r = 0.53, R^2^ = 0.26, ANOVA *p*-value <0.001.

## 4 Discussion

The current study aimed to examine the relationship between contraception use, pregnancy planning, and the risk of PND among Omani mothers. While the majority of the participating mothers reported planned pregnancies, a considerable number of them (26.0%) were unplanned pregnant. As expected, a significantly higher proportion of mothers with unplanned pregnancies were non-users of contraception. The findings underscore the intricate interplay between socio-demographic factors and pregnancy planning as well as contraception use among the study participants. Specifically, mothers with a monthly income of 1,000 OMR or lower and those who live in core families tend more not to use contraception. Furthermore, significantly higher prevalence of unplanned pregnancies was reported in mothers with lower gravida and primiparous status, highlighting the importance of considering pregnancy intention in maternal healthcare and family planning interventions. However, neither pregnancy planning nor contraception use were found to be associated with perinatal depression in our sample.

A more comprehensive understanding of the intricate interplay between socio-demographic factors, pregnancy planning, contraception use, and perinatal depression is crucial. The study identified the economic status as one of the significant factors associated with lower contraception use and higher prevalence of unplanned pregnancies. Much of the existing evidence highlights the relation between these variables indirectly. For example, a study by Kovacheva et al. in Spain revealed that women with suspected depression often faced economic challenges, such as unemployment, limited job opportunities, and inadequate maternity leave benefits (30). This aligns with the study's findings regarding the impact of economic resources on contraception use and perinatal depression risk. Similarly, research by Cena et al. revealed a strong correlation between economic disadvantages and the prevalence of perinatal depression in Italy (31). Moreover, Ahmed et al. conducted a study in Egypt focusing on PPD in primary care settings, involving 257 mothers (16). Their study revealed a notable prevalence of PPD at 33.5% among mothers from economically disadvantaged families, highlighting the substantial impact of socioeconomic factors on maternal mental health outcomes. Furthermore, our research indicated that employed women were more inclined to have planned pregnancies, underscoring the significance of financial stability and job security in family planning decisions. Family structure also emerged as a contributing factor, with a higher prevalence of core families observed in the planned pregnancy cohort. These findings align with existing literature emphasizing the influence of socioeconomic and familial factors on reproductive choices. Notably, monthly income showed a trend towards significance in relation to planned pregnancies, suggesting that women with moderate incomes were more likely to engage in pregnancy planning. The factors identified in our study, along with those in previous (16, 30, 31) underscore the substantial role of socioeconomic status in shaping contraception use, pregnancy planning decisions, and subsequently influencing the risk of perinatal depression. These findings emphasize the critical need for targeted interventions and support systems in maternal healthcare to address these intricate relationships effectively.

The findings identified an association between gravida, parity, and planned pregnancies suggesting that primiparous women were more likely to have planned pregnancies. Thus, emphasizing the impact of prior pregnancies and childbirth experiences on family planning decisions. Similar findings highlighted that primiparous women, experiencing their first pregnancy, are more likely to have planned pregnancies compared to multiparous women who have had previous childbirth experiences (32, 33). Furthermore, risk for PPD was significantly associated with antenatal depression risk in our sample, underscoring the continuity of mental health challenges across the perinatal period (34). The number of pregnancies also played a significant role, with women having four or five pregnancies showing increased risk of antenatal depression, which is in line with other studies’ findings such as Yu et al. (35). Additionally, employment status was a significant predictor of antenatal depression risk in our sample, emphasizing the potential influence of work-related stressors on maternal mental health (35). These findings underscore the need for workplace support and tailored mental health interventions for pregnant women. In the context of PPD risk, higher scores of antenatal depression risk (according to EPDS score) were the most influential predictor, reinforcing the interconnection between mental health states during the perinatal period. The number of pregnancies, specifically having only one compared to others, approached significantly as a predictor of PPD, indicating the potential impact of unique challenges faced by primiparous women. Furthermore, as studies have shown that a history of PPD predicts depression in subsequent pregnancies, it is important to consider past experiences when assessing the risk of PPD (36).

Research has indicated that unplanned pregnancies may be predictive of antenatal depression (22) and PPD, regardless of a woman's emotional stance toward pregnancy (6). However, the current study did not find a significant association between pregnancy planning and perinatal depression within the sample. Similarly, significant correlation between contraception use and perinatal depression were not observed. The occurrence of unplanned pregnancies often stems from inadequate contraception use. It has also been noted that there exists a complex relationship between contraception use and the risk of PPD. For instance, preconception depression could lead to suboptimal contraceptive utilization (37), while certain contraceptives have been linked to PPD (38). The lack of association between contraception use and perinatal depression in the current study's findings could be related to several factors. The demographic and reproductive health profiles of the sample may differ significantly from those in other studies, such as variations in age, parity, and socioeconomic status (39). Additionally, the type of contraception used can influence the association with depression, and our sample might have included a diverse range of contraceptive methods, potentially diluting any specific effects (40). Hormonal sensitivity is another consideration, as some women may be more sensitive to hormonal transitions associated with certain types of contraception (41, 42). Still, our sample might not have had a sufficient number of such women to detect a significant association. Methodological differences, including the measurement tools and timing of assessments, could also impact the observed associations. Furthermore, regional and cultural factors, such as access to mental health services, social support, and healthcare provider discussions about contraception (39), could influence the relationship between contraception use and perinatal depression in our study setting. Furthermore, the absence of an association between contraception use and perinatal depression in the sample could be attributed to a combination of these factors, highlighting the need for further research to explore these differences and better understand the complex interactions between contraception use, hormonal sensitivity, and mental health outcomes in diverse populations. Moreover, further research is essential to elucidate the potential predictive role of contraception use in perinatal depression, given previous studies have established a connection between unplanned pregnancies and perinatal depression (8, 9). These findings underscore the importance of family planning, tailored mental health interventions, and screening for pregnancy intentions in primary healthcare settings to support maternal mental well-being. Furthermore, the results suggest that women experiencing unplanned pregnancies should receive early detection and preventive measures for PPD, irrespective of their emotional stance, highlighting the necessity for targeted interventions.

### 4.1 Limitations

While the current study has many methodological advantages, it has some limitations as well. Some of the strengths could be the use of a prospective longitudinal study design which allowed for the examination of PND risk over time (6), use of a standardized assessment tool (EPDS), and collection of sociodemographic data self-reported by participants which is essential for understanding the contextual factors influencing PND risk. Nevertheless, possible limitations of the current study could be related to the recruitment of participants from only three major healthcare centers in a specific region of Oman which might have introduced a sampling bias (43). In addition, convenience sampling may not accurately represent the broader population, potentially limiting the generalizability of the findings. Also, reliance on self-reported data, including sociodemographic information and mental health status, may introduce response bias or social desirability bias. Therefore, objective measures or additional validation methods could enhance the reliability of the data collected. It is also important to consider the timing of data collection, both prenatally and postpartum, and its potential impact on the mental health status of the participating mothers.

## 5 Conclusions

This study explored the complex relationship between contraception use, pregnancy planning, and the risk of perinatal depression (PND) among Omani mothers. While a significant portion of mothers experienced unplanned pregnancies, highlighting the importance of considering pregnancy intention in maternal healthcare and family planning interventions, neither pregnancy planning, nor contraception use were directly associated with perinatal depression in the sample. The research identified economic status as a significant factor influencing contraception use and the prevalence of unplanned pregnancies, emphasizing the critical role of socioeconomic factors in reproductive choices and maternal mental health outcomes.

These insights are especially relevant in the Omani context, offering valuable information for healthcare providers, policymakers, and researchers. Comprehensive strategies aimed at reducing unplanned pregnancies and workplace support for pregnant women are crucial components in mitigating the prevalence of PND risk. Integrating routine mental health screening into antenatal care can facilitate early detection and targeted interventions, contributing to improved maternal mental well-being. Future research and interventions should explore targeted strategies to support women with specific risk factors, fostering optimal mental well-being throughout the perinatal period.

## Data Availability

The raw data supporting the conclusions of this article will be made available by the authors, without undue reservation.
